# Synergy between chemotherapeutic agents and CTLA-4 blockade in preclinical tumor models

**DOI:** 10.1007/s00262-013-1451-5

**Published:** 2013-07-20

**Authors:** Maria Jure-Kunkel, Gregg Masters, Emel Girit, Gennaro Dito, Francis Lee, John T. Hunt, Rachel Humphrey

**Affiliations:** 1 Bristol-Myers Squibb Company, PO Box 4000, Princeton, NJ 08543 USA; 2 MethylGene Inc., 7150 Frederick-Banting, Suite 200, Montreal, QC H4S 2A1 Canada; 3 MethylGene US, 125 Village Blvd., Princeton, NJ 08540 USA

**Keywords:** Chemotherapy, CTLA-4, Preclinical, Synergy, Tumor model

## Abstract

Ipilimumab, a cytotoxic T-lymphocyte antigen-4 (CTLA-4) binding agent, has proven to be an effective monotherapy for metastatic melanoma and has shown antitumor activity in trials when administered with other therapeutic agents. We hypothesized that the combination of ipilimumab with chemotherapeutic agents, such as ixabepilone, paclitaxel, etoposide, and gemcitabine, may produce therapeutic synergy based on distinct but complementary mechanisms of action for each drug and unique cellular targets. This concept was investigated using a mouse homolog of ipilimumab in preclinical murine tumor models, including SA1N fibrosarcoma, EMT-6 mammary carcinoma, M109 lung carcinoma, and CT-26 colon carcinoma. Results of CTLA-4 blockade in combination with one of various chemotherapeutic agents demonstrate that synergy occurs in settings where either agent alone was not effective in inducing tumor regression. Furthermore, when combined with CTLA-4 blockade, ixabepilone, etoposide, and gemcitabine elicited prolonged antitumor effects in some murine models with induction of a memory immune response. Future investigations are warranted to determine which specific chemo-immunotherapy combinations, if any, will produce synergistic antitumor effects in the clinical setting.

## Introduction

A complex and multifaceted interplay exists between the immune system and cancer. Innate and adaptive immune responses function to protect the host by attempting to mediate rejection of the tumor, and conversely, the immune system can also facilitate tumor progression by secreting factors that support tumor growth and immune escape, suppressing effective antitumor immunity. Furthermore, during cancer progression, tumor cells can develop multiple strategies to evade immune detection and destruction [1, 2]; thus, agents that modulate immune function are attractive therapeutic options to generate and expand robust and effective antitumor immune responses.

A vastly improved understanding of the mechanisms and pathways that govern immune regulation has led to the evaluation of novel therapeutic approaches targeting specific immune receptors/ligands within these pathways. Cytotoxic T-lymphocyte antigen-4 (CTLA-4) is one such receptor, and its role as a key negative regulator of T-cell responses gives it the potential as a target for therapy in multiple cancer types [3, 4].

One of the key events in the initiation of adaptive immunity is the antigen-specific activation of naïve T cells, a process which is controlled by a precise balance of stimulatory and inhibitory regulatory signals. Multiple signals are required for effective T-cell activation [5], which is initiated by the engagement of the major histocompatibility antigen complex. After activation, CTLA-4, a member of the immunoglobulin family and a homolog of CD28, is expressed on the surface of T cells [6]. CTLA-4, which has a higher affinity for binding B7 molecules than does CD28, curtails T-cell activation and proliferation by various mechanisms, including competitive inhibition of CD28, delocalization of protein kinase C-theta and CARMA1 from the immune synapse, transendocytosis of B7, and modulation of regulatory T-cell (Treg) function [7–15].

Preclinical investigations in in vivo systems have confirmed the key role of CTLA-4 in immune regulation and immunotherapy, demonstrated by the phenotype of CTLA-4 knockout mice, which develop a lethal lymphoproliferative phenotype at a young age [16, 17]. Moreover, anti-CTLA-4 monoclonal antibody (mAb) therapy in preclinical cancer models produced antitumor activity, both as monotherapy and in combination with other therapeutic modalities [3, 18–21], providing the rationale for clinical development of human monoclonal antibodies that target CTLA-4.

Two fully human mAbs that bind CTLA-4, tremelimumab (CP-675,206, Pfizer, New York, NY) and ipilimumab [22] (Yervoy™, Bristol-Myers Squibb, Princeton, NJ), have been in clinical development over the past decade [23, 24], and both agents have shown activity in inducing tumor regression in clinical studies [25, 26]. Notably, ipilimumab has been approved in over 40 countries at a dose of 3 mg/kg for the treatment of unresectable or metastatic melanoma, following the results of a phase III study in advanced melanoma, in which ipilimumab improved the overall survival relative to patients given melanoma vaccine glycoprotein 100 monotherapy, with a side-effect profile that was inflammatory in nature, consistent with the agent’s immune-based mechanism of action [24]. Ipilimumab also demonstrated improved survival and tolerability when administered with dacarbazine, a chemotherapeutic agent, in a phase III trial in chemotherapy-naïve patients with metastatic melanoma [27].

Chemo-immunotherapy is a novel approach for the treatment of cancer that combines drugs that directly kill tumor cells with interventions that modulate host immune responses to the tumor. Preclinical and clinical evidence suggests that chemotherapy may induce or support immunity against tumor cells by various mechanisms. Chemotherapy-induced cell death may generate tumor antigens to be presented by APCs, creating a “polyvalent” tumor-cell vaccine in situ. Additionally, cytotoxic treatments may distort the tumor architecture, thus facilitating the penetration of the immunotherapeutic agents and the expanded immune population [28–30]. The mechanism by which a given chemotherapy impacts the immune system may be different from another chemotherapy agent. As such, combining immunotherapeutic strategies with more traditional therapies, such as chemotherapeutic agents, vaccines, and radiotherapy, is of particular clinical interest in the treatment of cancer, but it remains to be seen which combinations will produce synergistic antitumor effects in the clinic. In addition to the potential for synergy, combining immunotherapy with chemotherapy has been proposed as a mechanism to overcome chemotherapeutic resistance, which is a critical barrier to effective treatment in some tumor types [31].

In the presented studies, we describe preclinical evidence of synergy between CTLA-4 blockade and chemotherapeutic agents in various murine tumor models of fibrosarcoma and cancers of the mammary gland, lung, and colon. The chemotherapeutic agents tested in the study, including ixabepilone, paclitaxel, etoposide, and gemcitabine, exhibit distinct mechanisms for antitumor activity and represent common therapeutic options. Although the studies herein employed a single schedule of drugs used at optimal dose (OD) and therefore are not designed for direct extrapolation to human malignancies—the antitumor effects observed in these preclinical models provide the rationale for further clinical investigation of these and other chemo-immunotherapy approaches for the treatment of cancer.

## Materials and methods

### Animals

Eight- to 12-week-old female BALB/c (Harlan, Indianapolis, IN) and A/J mice (Jackson, Bar Harbor, MA, USA) comprised each cohort of 8–12 mice. The mice received food and water ad libitum and were maintained in a controlled environment according to the Association for Assessment and Accreditation of Laboratory Animal Care (AAALAC) International regulations. All animal studies have been approved by the appropriate ethics committee and have, therefore, been performed in accordance with the ethical standards laid down in the 1964 Declaration of Helsinki and its later amendments.

### Antibodies and chemotherapeutic agents

Since ipilimumab is specific to human CTLA-4, these experiments utilized an anti-mouse CTLA-4 mAb (anti-mCTLA-4 mAb) clone 4F10-UC10-11 at an OD of 20 mg/kg unless otherwise noted. Clone 4F10-UC10-11 was obtained from the American Type Culture Collection (ATCC, Manassas, VA, USA). Anti-mCTLA-4 mAb was produced and purified by Bristol-Myers Squibb (Protein Therapeutics Division, Hopewell, NJ, USA) and was certified to have <0.5 EU/mg endotoxin levels, >95 % purity, and <5 % high molecular weight species. Stock solutions of anti-mCTLA-4 mAb were kept at −80 °C and thawed at 4 °C prior to use. Polyclonal hamster IgG (Jackson ImmunoResearch, West Grove, PA, USA) was utilized as the control antibody. Dosing solutions of anti-mCTLA-4 mAb and hamster IgG control were prepared in sterile phosphate-buffered saline (pH 7.0). Antibodies used for immunostaining of tumor-draining lymph nodes (TDLN) were purchased from BD Biosciences (San Jose, CA, USA).

Chemotherapeutic agents employed included ixabepilone (8 mg/kg, Bristol-Myers Squibb, Princeton, NJ, USA), paclitaxel (24 mg/kg, Fondazione Michelangelo, Milan, Italy), etoposide (40 mg/kg, LC Laboratories, Woburn, MA, USA), and gemcitabine (120 mg/kg, Eli Lilly, Indianapolis, IN, USA). Of note, these agents were chosen because they are broadly utilized clinically as standards of care across a wide spectrum of solid tumors.

Anti-mCTLA-4 mAb, ixabepilone, paclitaxel, or gemcitabine were given every 4 days for 3 doses by intraperitoneal injection. Etoposide was administered intravenously every 7 days for 3 doses. All drugs were administered in an optimal schedule and dose specific to each tumor model as determined in the preliminary experiments (data not shown). To help mitigate the potential for chemotherapy to affect T-cell function or viability, anti-mCTLA-4 mAb was given 1 day after chemotherapy dosing (unless otherwise noted in tables or figure legends).

### Tumor models

SA1N fibrosarcoma, EMT-6 mammary carcinoma, M109 lung carcinoma, and CT-26 colon carcinoma tumor lines used in this study were maintained in vitro. Cell suspensions were implanted in the subcutaneous space of the flank of mice. In some studies, mice that showed complete tumor regression after therapy were rechallenged with a lethal dose of tumor cells to determine the level of immune protection.

Efficacy studies were performed with each of the tumor models. Antibodies and chemotherapeutic agents were administered at OD; dosing schedules and routes for administration are shown for each study in Table 1 or described in figure legends. Each treatment regimen consisted of cohorts containing 8–12 mice. Tumor size and body weights were measured twice weekly. Tumor size (measured as mm^3^) was calculated by multiplying the tumor length by the square of the tumor width and then dividing by 2. Treatments were initiated when subcutaneous tumors reached a median size between 125 and 225 mm^3^ (established model; SA1N fibrosarcoma and CT-26 colon carcinoma models) or prior to detection (initiation model; EMT-6 mammary carcinoma and M109 lung carcinoma models), depending on the model’s sensitivity to chemotherapy. In the established model, antitumor activity, defined as percentage tumor growth inhibition, was calculated with the formula $\% \,{\text{Tumor Growth Inhibition }}\,\left( {\% {\text{TGI}}} \right) = 100 - \left[ {\left( {{\text{Tt}}/{\text{To}}} \right)/\left( {{\text{Ct}}/{\text{Co}}} \right)} \right]/ 100 - \left( {{\text{Ct}}/{\text{Co}}} \right)$, where Tt = median tumor size of treated group at the end of treatment, To = median tumor size of treated group at treatment initiation, Ct = median tumor size of control group at the end of treatment, and Co = median tumor size at treatment initiation. Since there is no baseline for tumor volume in the initiation model, and therefore %TGI cannot be calculated, we reported the percentage of tumor-free mice at the end of each experiment. The tumor response endpoint was expressed as tumor growth delay (T–C value), calculated as the difference in time (days) between the treated (T) and control (C) groups for the tumor to reach a predetermined target size. A delay in reaching target size by the treated groups of >1 times tumor volume doubling time was considered an active result. In the intravenous M109 lung carcinoma model, survival was the targeted endpoint. Therapeutic synergy was defined as an antitumor effect in which the combination of agents demonstrated significant superiority (*p* < 0.05) relative to the activity shown by each agent alone.

**Table 1:** Antitumor activity of CTLA-4 blockade in combination with chemotherapies in tumor models

Tumor model (site of tumor cell implantation, host)	Tx	Schedule (days post-tumor implant)	% TGI	T–C (days)	% CR or % tumor-free mice (# per total mice)	Best combination effect
*Ixabepilone or paclitaxel in combination with anti-mCTLA-4 mAb*						
SA1N fibrosarcoma (SC, A/J mice)^a,b^	CTLA-4 mAb	Day 12, 16, 20	79	7	25 (2/8)	
	Ixa	Day 11, 15, 19	83	7	0 (0/8)	
	Ixa + CTLA-4 mAb	Day 11, 15, 19 Day 12, 16, 20	112	>95	71.4 (5/7)	Therapeutic synergy
	CTLA-4 mAb	Day 11, 15, 19	79	7	25 (2/8)	
	Pac	Day 10, 14, 18	0	0	0 (0/8)	
	Pac + CTLA-4 mAb	Day 10, 14, 18 Day 11, 15, 19	112	>95	87.5 (7/8)	Therapeutic synergy
EMT-6 mammary Ca (SC, Balb/c mice)	CTLA-4 mAb	Day 4, 8, 12	N/A	29	40 (4/10)	
	Ixa	Day 3, 7, 11	N/A	19	20 (2/10)	
	Ixa + CTLA-4 mAb	Day 3, 7, 11 Day 4, 8, 12	N/A	>37	100 (10/10)	Therapeutic synergy
	Pac	Day 3, 7, 11	N/A	0	0 (0/10)	
	Pac + CTLA-4 mAb	Day 3, 7, 11 Day 4, 8, 12	N/A	37	40 (4/10)	
M109 lung Ca (SC, Balb/c mice)^a^	CTLA-4 mAb	Days 4, 8, 12	N/A	4	0 (0/10)	
	Ixa	Days 3, 7, 11	N/A	>79	50 (5/10)	
	Ixa + CTLA-4 mAb	Days 3, 7, 11 Days 4, 8, 12	N/A	>79	80 (8/10)	Rejection of tumor rechallenge (75 % mice with combination vs. 20 % of mice treated with Ixa alone)
	Pac	Days 3, 7, 11	N/A	7	0 (0/10)	
	Pac + CTLA-4 mAb	Days 3, 7, 11 Days 4, 8, 12	N/A	11	20 (2/10)	Therapeutic synergy
CT-26 colon Ca (SC, Balb/c mice)	CTLA-4 mAb	Days 5, 9, 13	92	17	20 (2/10)	
	Ixa	Days 4, 8, 12	26	0	0 (0/10)	
	Ixa + CTLA-4 mAb	Days 4, 8, 12 Days 5, 9, 13	103	>17	70 (7/10)	Therapeutic synergy
	Pac	Days 4, 8, 12	2	0	0 (0/10)	
	Pac + CTLA-4 mAb	Days 4, 8, 12 Days 5, 9, 13	103	>17	50 (5/10)	
*Etoposide in combination with anti-mCTLA-4 mAb*						
SA1N fibrosarcoma (SC, A/J mice)	CTLA-4 mAb^c^	Days 15, 18, 22	78	23	12.5 (1/8)	
	Etop	Days 14, 21, 28	61	14	0 (0/8)	
	Etop + CTLA**-**4^c^ mAb	Days 14, 21, 28 Days 15, 18, 22	105	64	62.5 (5/8)	Therapeutic synergy
M109 lung Ca (IV, Balb/c)	CTLA-4 mAb	Days 5, 9, 13	N/A	1	N/A	
	Etop^d^	Days 4, 11, 18	N/A	2.5	N/A	
	Etop + CTLA-4 mAb^d^	Days 4, 11, 18 Days 5, 9, 13	N/A	11.5	N/A	Therapeutic synergy
CT-26 colon Ca (SC, Balb/c)^a^	CTLA-4 mAb	Days 9, 13, 17	22	0	0 (0/8)	
	Etop^d^	Days 8, 15, 22	82	11	12.5 (1/8)	
	Etop + CTLA-4 mAb^d^	Days 8, 15, 22 Days 9, 13, 17	101	63	50 (4/8)	Therapeutic synergy (4/4 mice rejected tumor rechallenge)
*Gemcitabine in combination with anti-mCTLA-4 mAb*						
SA1N fibrosarcoma (SC, A/J mice)^a^	CTLA-4 mAb^c^	Days 15, 18, 22	78	23	12.5 (1/8)	
	Gem	Days 14, 18, 22	57	11	0 (0/8)	
	Gem + CTLA-4 mAb^c^	Days 14, 18, 22 Days 15, 18, 22	86	23	25 (2/8)	No therapeutic synergy
M109 lung Ca (IV, Balb/c mice)^a^	CTLA-4 mAb	Days 5, 9, 13	N/A	3.5	N/A	
	Gem	Days 4, 8, 12	N/A	11.5	N/A	
	Gem + CTLA-4 mAb	Days 4, 8, 12 Days 5, 9, 13	N/A	36.5	N/A	Therapeutic synergy
CT-26 colon Ca (SC, Balb/c mice)^a^	CTLA-4 mAb	Days 9, 13, 17	22	0	0 (0/8)	
	Gem	Days 8, 12, 16	85	11	25 (2/8)	
	Gem + CTLA-4 mAb	Days 8, 12, 16 Days 9, 13, 17	103	85	62.5 (5/8)	Therapeutic synergy (5/5 mice rejected tumor rechallenge vs. 2/2 for Gem alone)

*CTLA*-*4 mAb* anti-mouse CTLA-4 monoclonal antibody, *Ca* carcinoma, *CR* complete regression of tumor, *Etop* etoposide, *Gem* gemcitabine, *Ixa* ixabepilone, *N/A* not applicable, *SC* subcutaneous, *T*–*C* number of days for treated group to reach target size—number of days for control group to reach target size, *%TGI* % tumor growth inhibition calculated on the last measurement for control group, *Tx* treatment

^a^Representative of two independent studies

^b^One CR, which was non-treatment related, was observed in the control group in the SA1N tumor model

^c^Anti-mCTLA-4 mAb was administered at a dose of 10 mg/kg

^d^Etoposide was administered at a dose of 50 mg/kg

### In vivo cytotoxic cell assay

To examine in vivo cytotoxicity, mice bearing subcutaneous CT-26 colon tumors were treated with anti-mCTLA-4 mAb and each chemotherapeutic agent (individually or in combination) as described. Two and 7 days after the final treatment, mice (*n* = 5/group) were injected with carboxyfluorescein diacetate succinimidyl ester (CFSE)-labeled syngeneic splenocytes pulsed with CT-26-specific peptides (peptide AH-1, (H) SPSYVYHQF (OH), Sigma Genosys; 2.5 μM CFSE) or left unpulsed as a control (0.25 μM CFSE). Eighteen hours later, mice were euthanized, spleens were removed, and splenocytes were isolated and resuspended in phosphate-buffered saline (PBS). Fluorescence of the cell suspensions was measured by flow cytometry, and cytolytic activity was determined by measuring the ratio of CFSE-labeled cells (CFSE high = peptide pulsed, CFSE low = not pulsed).

### Immunophenotyping of TDLN

To analyze the composition of TDLN in the CT-26 colon carcinoma and M109 lung carcinoma tumor models, TDLN were collected and cell suspensions were prepared with a hand-held homogenizer. Cells were counted and diluted into staining buffer (PBS, pH 7.0 plus 1 % fetal calf serum and 0.1 % sodium azide) at a concentration of 1 × 10^7^cells/mL. Cells were stained with fluorescent-labeled antibodies for 45 min on ice, followed by two washes in staining buffer. Cells were fixed in 0.1 % formaldehyde and then subjected to flow cytometry analyses.

## Results

### Antitumor activity of CTLA-4 blockade in combination with microtubule-stabilizing agents, ixabepilone and paclitaxel

**Fig. 1:**
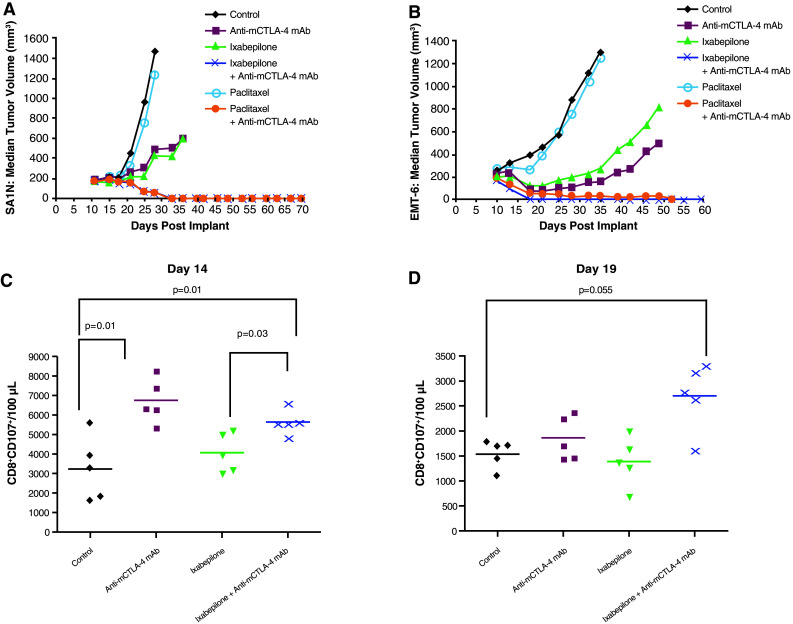

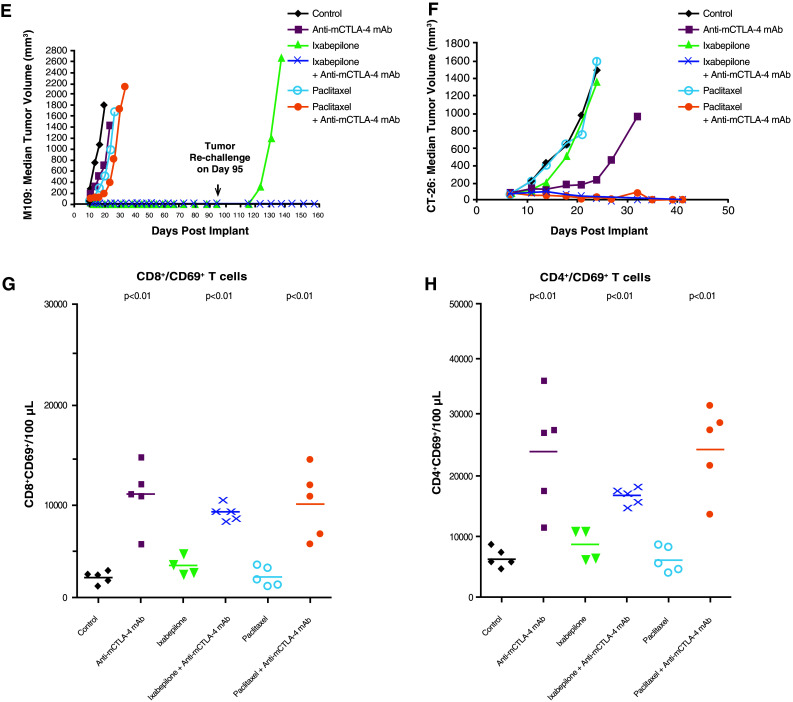
Therapeutic synergy observed with CTLA-4 blockade in combination with ixabepilone and paclitaxel in tumor models. In the SA1N fibrosarcoma model (**a**), combination of anti-mCTLA-4 mAb with either ixabepilone or paclitaxel resulted in therapeutic synergy, with the majority of mice displaying substantially delayed tumor growth over time. In the EMT-6 mammary carcinoma model (**b**), combination of anti-mCTLA-4 mAb with ixabepilone yielded synergistic effects over time, resulting in complete regression of tumors on Day 18; anti-mCTLA-4 mAb paired with paclitaxel improved antitumor activity without achieving a synergistic effect. Anti-mCTLA-4 mAb in combination with ixabepilone expanded T lymphocytes with cytolytic function by Day 19 (CD8^+^CD107^+^), supporting synergistic efficacy in the EMT-6 mammary carcinoma model (**c**, **d**). In the M109 lung carcinoma model (**e**), tumor-free mice previously treated with ixabepilone monotherapy or in combination with anti-mCTLA-4 mAb were rechallenged on Day 95 with live tumor cells. The majority of mice (75 %) treated with the combination of anti-mCTLA-4 mAb and ixabepilone rejected the tumor rechallenge, suggestive of a memory immune response. In the CT-26 colon carcinoma model (**f**), treatment of mice with anti-mCTLA-4 mAb and either ixabepilone or paclitaxel resulted in synergy between CTLA-4 blockade and these chemotherapeutic agents. Expansion of activated T cells (CD8^+^/CD69^+^ and CD4^+^/CD69^+^) was observed with anti-mCTLA-4 mAb alone and in combination with either ixabepilone or paclitaxel (**g**, **h**)

In the SA1N fibrosarcoma model, use of ixabepilone in combination with anti-mCTLA-4 mAb demonstrated therapeutic synergy, yielding 112 % TGI, with 71.4 % (*n* = 5/7) of the animals displaying complete tumor regression (Table 1; Fig. 1a). Although paclitaxel monotherapy had no therapeutic effect, addition of anti-mCTLA-4 mAb to paclitaxel treatment yielded complete responses in 87.5 % (*n* = 7/8) of the animals in the SA1N fibrosarcoma model (Table 1; Fig. 1a) [32]. Both combination regimens enhanced the antitumor effect of each monotherapy and significantly delayed tumor growth to target size, resulting in therapeutic synergy.

In the EMT-6 mammary carcinoma mouse model, the combination of ixabepilone and anti-mCTLA-4 mAb resulted in a synergistic therapeutic effect, inducing complete regressions in 100 % (*n* = 10/10) of the treated mice (Table 1; Fig. 1b). Paclitaxel alone did not show an antitumor effect in the EMT-6 mammary carcinoma mouse model (Table 1; Fig. 1b); furthermore, mice treated with both paclitaxel and anti-mCTLA-4 mAb blockade displayed improved antitumor activity without achieving a synergistic effect (Table 1; Fig. 1b). Ixabepilone in combination with anti-mCTLA-4 mAb expanded T lymphocytes with cytolytic function by Day 19 (CD8^+^CD107^+^) (Fig. 1c, d) [32], an effect that was not observed with paclitaxel in combination with anti-mCTLA-4 mAb, consistent with the distinct therapeutic outcome observed with these combinations.

Although treatment of M109 lung carcinoma mice with ixabepilone alone produced effective inhibition of tumor growth with 50 % of mice tumor-free following the initial transplantation, therapy with either CTLA-4 blockade alone or paclitaxel alone failed to demonstrate the antitumor activity (Table 1; Fig. 1e). Combination of ixabepilone or paclitaxel with anti-mCTLA-4 mAb resulted in 80 % (8/10 mice) and 20 % (2/10 mice) tumor-free mice, respectively, demonstrating enhanced antitumor activity compared with either chemotherapeutic agent alone (Table 1; Fig. 1e). To investigate whether the addition of CTLA-4 blockade to ixabepilone modulated the immune response to tumors, mice which were previously treated with ixabepilone or ixabepilone plus anti-mCTLA-4 mAb that were tumor-free were rechallenged on Day 95 with M109 tumor cells subcutaneously. A cohort of untreated mice served as controls, which had tumors that grew progressively (Fig. 1e). Conversely, the majority (75 %; *n* = 6/8) of mice treated with the combination of ixabepilone and anti-mCTLA-4 mAb rejected a subsequent tumor rechallenge on Day 95, as compared with only 20 % of mice treated with ixabepilone alone (Table 1; Fig. 1e).

Finally, in the CT-26 colon carcinoma model, the combination of ixabepilone or paclitaxel with anti-mCTLA-4 mAb resulted in effective tumor rejection, with 50–70 % (*n* = 5/10 and 7/10, respectively) of mice displaying complete tumor regression (Table 1; Fig. 1f). Additionally, expansion of activated T cells (CD8^+^ and CD4^+^) was observed with anti-mCTLA-4 mAb alone and in combination with either ixabepilone or paclitaxel (Fig. 1g, h) [32].

### Antitumor activity of etoposide in combination with anti-mCTLA-4 mAb

**Fig. 2:**
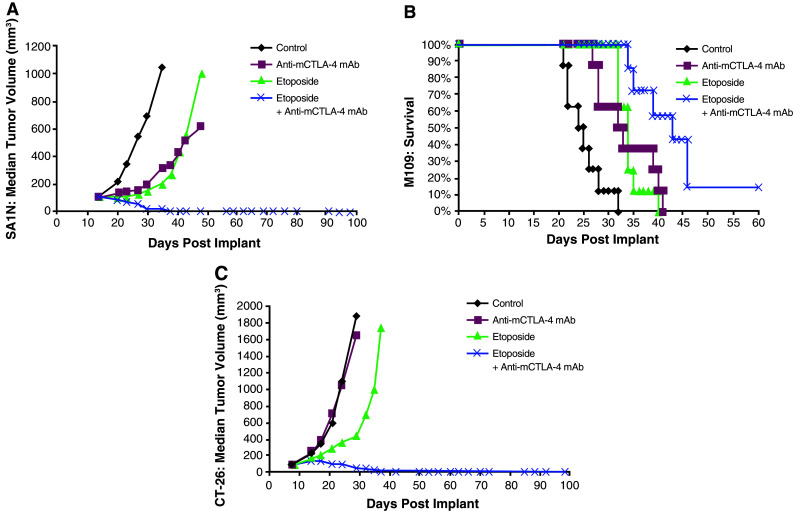
Therapeutic synergy observed with CTLA-4 blockade in combination with etoposide in tumor models. In the SA1N fibrosarcoma model (**a**), M109 lung carcinoma model (**b**), and CT-26 colon carcinoma model (**c**), therapeutic synergy was observed with the administration of anti-mCTLA-4 mAb in combination with etoposide relative to the treatment with either monotherapy. Efficacy was evaluated either by tumor volume (**a**, **c**) or by survival measurement (**b**)

Therapeutic synergy was observed with etoposide and anti-mCTLA-4 mAb in the SA1N fibrosarcoma, M109 lung carcinoma, and CT-26 colon carcinoma murine models (Table 1; Fig. 2). In the SA1N fibrosarcoma model, the combination produced complete tumor regression in the majority of mice (62.5 %; *n* = 5/8) (Table 1; Fig. 2a). Additionally, significant prolongation of survival was observed in the M109 lung metastasis model when this combination was administered starting 4 days after intravenous tumor cell inoculation, which resulted in synergistic effects (Table 1; Fig. 2b).

**Fig. 3:**
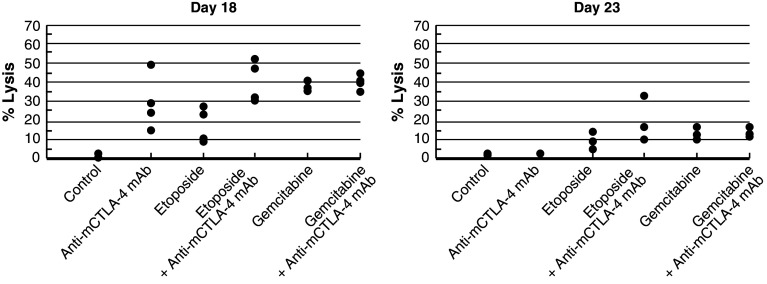
In vivo cytotoxic activity toward a CT-26 tumor antigen. In the CT-26 colon carcinoma mouse model, anti-mCTLA-4 mAb was administered at a dose of 20 mg/kg on Days 8, 12, and 16. Etoposide was administered at a dose of 50 mg/kg on Days 7, 14, and 21, whereas gemcitabine was administered at a dose of 120 mg/kg on Days 7, 11, and 15. In vivo cell kill was determined on Days 18 and 23 post-implant. One day prior to analysis, a 50:50 mixture of peptide-pulsed ([H] SPSYVYHQF [OH], Sigma Genosys) and peptide non-pulsed carboxyfluorescein diacetate succinimidyl ester (CSFE)-labeled splenocytes from naive BALB/c donors were adoptively transferred via IV tail injection into treated animals; 24 h later, spleens were removed and analyzed via flow cytometry to determine the percent cell kill of peptide-pulsed cells. In vivo cytotoxicity against a CT-26 tumor antigen showed a slight increase in the CTLA-4 blockade and etoposide combination group without reaching statistical significance versus anti-mCTLA-4 mAb alone. At these time points, there were no significant enhancements of the cytotoxic activity against a CT-26 tumor antigen with the combination of anti-mCTLA-4 mAb and gemcitabine when compared with either treatment alone

In the CT-26 colon carcinoma model, neither etoposide nor anti-mCTLA-4 mAb alone were active at OD levels, but their combination exhibited synergistic effects (Table 1; Fig. 2c). Mice that reached complete regression or naïve mice were rechallenged with 1 × 10^6^ CT-26 cells subcutaneously on Day 77 post-tumor cell implantation. Although all naïve mice developed tumors that grew progressively, each of the mice treated with etoposide in combination with anti-mCTLA-4 mAb (*n* = 4) rejected tumor rechallenge, leading the authors to interpret that combination of CTLA-4 blockade plus etoposide generated a memory immune response (Table 1). In vivo cytotoxicity against a CT-26 tumor antigen showed a slight increase in the combination group versus anti-mCTLA-4 mAb without reaching statistical significance (Fig. 3).

### Antitumor activity of gemcitabine in combination with anti-mCTLA-4 mAb

**Fig. 4:**
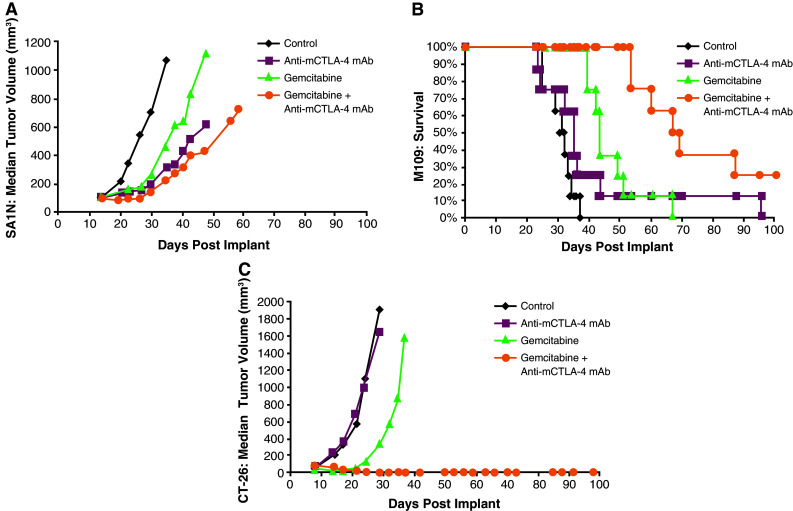
Therapeutic synergy observed with CTLA-4 blockade in combination with gemcitabine in tumor models. In the SA1N fibrosarcoma model (**a**), the combination of anti-mCTLA-4 mAb with gemcitabine did not produce therapeutic synergy. In the M109 lung carcinoma model (**b**) and CT-26 colon carcinoma model (**c**), therapeutic synergy was observed with administration of anti-mCTLA-4 mAb in combination with gemcitabine relative to the treatment of mice with either monotherapy

Co-administration of gemcitabine and anti-mCTLA-4 mAb demonstrated synergy in the CT-26 colon carcinoma and M109 lung carcinoma models, but not in the SA1N fibrosarcoma model (Table 1; Fig. 4). In the SA1N fibrosarcoma model, anti-mCTLA-4 mAb alone and gemcitabine alone showed modest activity but the combination of both agents in this model did not result in therapeutic synergy (Table 1; Fig. 4a). In the M109 lung metastasis tumor model, OD levels of gemcitabine and anti-mCTLA-4 mAb in combination resulted in therapeutic synergy with animals demonstrating increased survival, relative to animals treated with either agent alone (Table 1; Fig. 4b). In the CT-26 colon carcinoma model, this combination resulted in synergistic effects, with 62.5 % (*n* = 5/8) of animals displaying complete regressions (Table 1; Fig. 4c); however, a lack of synergistic effects was observed in the CT-26 colon carcinoma model when a sequential dosing regimen was explored, where 3 doses of gemcitabine were administered first (120 mg/kg) followed by administration of 3 doses of anti-mCTLA-4 mAb (20 mg/kg) (data not shown).

Similar to the experiments performed with etoposide and ixabepilone as described previously, mice that had reached complete regression in the CT-26 colon carcinoma were rechallenged with a lethal dose of tumor cells (1 × 10^6^ CT-26 cells) on Day 77 post-tumor implantation to determine the level of immune protection. Naïve mice were also included as a control in this assay, and as expected, they developed tumors that grew increasingly upon being challenged. However, all of the mice in the gemcitabine treatment group (*n* = 2) and all of the mice treated with gemcitabine plus anti-mCTLA-4 mAb (*n* = 5) that initially displayed complete regressions rejected subsequent tumor rechallenge (Table 1).

No significant enhancement of the cytotoxic activity against a CT-26 tumor antigen was observed with the combination of gemcitabine and anti-mCTLA-4 mAb when compared with either treatment alone, as measured in the in vivo cytotoxic cell assay (Fig. 3). However, the combination treatment modulated the composition of the TDLN cells. Specifically, gemcitabine in combination with anti-mCTLA-4 mAb increased the levels of activated (CD69^+^) CD4 and CD8 T cells while decreasing the number of myeloid-derived suppressor cells (MDSC; CD11b^+^GR1^+^) (Table 2).

**Table 2:** Effect of CTLA-4 blockade and gemcitabine on immune cells in tumor-draining lymph nodes following 3 doses

Immune cell populations	Control	CTLA-4 mAb	Gem	Gem + CTLA-4 mAb
% CD4^+^	40.6 ± 1.9	45.7 ± 3.5	48.8 ± 5.1 (*p* < 0.05)	48.1 ± 3.3 (*p* < 0.05)
% CD4^+^ CD69^+^ (activated CD4^+^ cells)	8.7 ± 1.0	14.2 ± 0.9 (*p* < 0.01)	9.5 ± 1.1	11.7 ± 0.5 (*p* < 0.01)
% CD8^+^	19.3 ± 0.5	18.6 ± 2.4	21.7 ± 2.1	20.5 ± 1.0
% CD8^+^ CD69^+^ (activated CD8^+^ cells)	4.2 ± 0.5	4.9 ± 0.2 (*p* < 0.05)	3.8 ± 0.4	4.8 ± 0.4 (*p* < 0.05)
% CD4^+^CD25^+^FoxP3 (regulatory T cells)	15.4 ± 0.7	20.9 ± 0.5 (*p* < 0.01)	12.1 ± 0.8 (*p* < 0.05)	15.1 ± 1.1
% CD11b^+^Gr1^+^ (myeloid-derived suppressor cells)	2.24 ± 0.5	2.45 ± 0.7	2.45 ± 0.8	1.45 ± 0.3 (*p* < 0.05)

*CTLA*-*4 mAb* anti-mouse CTLA-4 monoclonal antibody, *Gem* gemcitabine, *p* *p* value versus control

In the CT-26 colon carcinoma model, gemcitabine was administered at a dose of 120 mg/kg on Days 7, 11, and 15, and anti-mCTLA-4 mAb was administered at a dose of 20 mg/kg on Days 8, 12, and 16 post-tumor implant. On Day 17, 4 mice/group were sacrificed and tumor-draining lymph nodes were collected and processed for immunophenotypic analyses. Data are expressed as mean % ± standard deviation

### Tolerability of the combination therapy

In each of the murine tumor models tested, addition of anti-mCTLA-4 mAb following administration of each chemotherapeutic agent did not affect body weight loss above the levels observed with the chemotherapeutic agents alone (data not shown).

## Discussion

The preclinical findings described offer evidence that the combination of CTLA-4 blockade with various chemotherapeutic agents that exhibit different mechanisms of action, including ixabepilone, paclitaxel, etoposide, and gemcitabine, elicited synergistic antitumor activity in murine tumor models when administered concurrently. In data from experiments in multiple tumor models involving treatment with CTLA-4 blockade and/or chemotherapeutic agents, synergy was observed in settings where blockade with anti-mCTLA-4 mAb alone was ineffective or in models where the chemotherapeutic agent alone did not induce tumor regression.

In tumor models where CTLA-4 blockade demonstrated modest antitumor effects, such as in the SA1N fibrosarcoma, EMT-6 mammary carcinoma, and CT-26 colon carcinoma models, synergy was observed in combination with chemotherapy, independent of the intrinsic potency of the chemotherapeutic agent, which suggests that the chemotherapeutic agents tested in this study may potentiate the effect of anti-mCTLA-4 mAb in settings where CTLA-4 blockade may be efficacious. Conversely, synergy with etoposide and gemcitabine was also observed in the M109 lung carcinoma model in which CTLA-4 blockade was inactive, even when these chemotherapeutic agents demonstrated modest effect as monotherapy. Nevertheless, the limitations of the present study preclude a definitive conclusion regarding the contributing factors responsible for the superior antitumor effect elicited by the combination treatments.

Although in some models we observed synergy and expansion of CD8^+^CD107^+^ T lymphocytes with CTLA-4 blockade in combination with ixabepilone, we did not see such an effect with paclitaxel. Because the mechanisms of action for ixabepilone and paclitaxel virtually overlap, the observed differences in efficacy (SA1N fibrosarcoma, EMT-6 mammary carcinoma, and M109 lung carcinoma models) and cytotoxic T cells (M109 lung carcinoma model) may be attributed to the inherent potency of ixabepilone in these models. On the other hand, while both ixabepilone and paclitaxel were inactive in the CT-26 colon carcinoma model, enhanced efficacy was observed in combination with anti-mCTLA-4 mAb, suggesting that perhaps minimum induction of tumor cell death was necessary in this model to prime an immune response and potentiate the effect of CTLA-4 blockade. However, a direct immunomodulatory effect by these agents cannot be eliminated [33–35].

In this study, treatment with anti-mCTLA-4 mAb increased the frequency of activated CD4^+^ and CD8^+^ T cells (CD69^+^), and this effect was not altered by the addition of chemotherapy. Expansion of activated T cells has been reported in clinical trials in melanoma with ipilimumab [36], which has been proposed to be the result of the pharmacodynamic activity of this compound. It was of interest then to observe that the addition of chemotherapy did not alter this effect in murine models, and, as such, it could be used as a candidate biomarker to evaluate how a combination partner may modulate this pharmacologic effect of ipilimumab in clinical settings.

Of note, tolerability to the chemotherapeutic agent was not altered with the addition of anti-mCTLA-4 mAb, indicating that the particular chemo-immunotherapy combinations tested did not affect the safety profile of the chemotherapeutic agent or produce overt toxicities in mice. Since adverse events resulting from therapy with CTLA-4 blocking antibodies in murine models are not predictive of adverse events in humans, definitive characterization of the tolerability of the combinations awaits further testing in the clinic.

Importantly, addition of anti-mCTLA-4 mAb to ixabepilone, etoposide, or gemcitabine resulted in the generation of a memory immune response in various tumor models, including the M109 lung carcinoma and CT-26 colon carcinoma models, as evidenced by the rejection of a secondary tumor challenge. It is increasingly recognized that chemotherapy may evoke an antitumor immune response, and that effect may be responsible for their efficacy in clinical settings [29]. However, chemotherapy alone may not produce the desirable effect of inducing immune memory, which may inhibit tumor relapse. Of note, CTLA-4 blockade has been shown previously to induce memory immune responses [32]. Our presented combination studies showed that chemotherapy supported, or at least did not blunt, the generation of a memory immune response. Furthermore, even in settings where chemotherapy yielded complete tumor regressions (e.g., ixabepilone in the M109 fibrosarcoma model), this effect was not sufficient to promote antitumor immunity since most of the mice that rejected tumor rechallenge also received anti-mCTLA-4 mAb (Table 1).

The results reported here demonstrate synergy between specific chemotherapeutic agents and CTLA-4 blockade in tumor regression. This is further supported by a recent study by Ariyan et al. [37] in which mice bearing a transplantable prostate tumor (TRAMPC2) treated with gemcitabine plus α-CTLA-4 experienced longer median survival (<125 days) than mice treated with gemcitabine monotherapy (72 days, *p* < 0.05). Furthermore, the study demonstrated that prolonged survival was associated with an accumulation of CD8 cells that are tumor-specific, whereas depletion of CD8 cells reduced the efficacy of this treatment [37]. A study by Lesterhuis et al. [38] demonstrated that a concurrent schedule of gemcitabine in combination with CTLA-4 blockade in two murine tumor models yields synergistic effects resulting in the induction of a potent antitumor immune response, which confirms our observations described in this manuscript. Of note, depletion analyses performed by Lesterhuis et al. [38] demonstrated that both CD4^+^ and CD8^+^ T cells are required for optimal therapeutic effect. Lastly, Wu et al. [39] have shown that CTLA-4 blockade in combination with cisplatin demonstrated improved antitumor activity versus each agent alone in a mouse model of mesothelioma. The combination therapy resulted in increased tumor infiltration of T cells and enhanced production of cytokines associated with cytotoxic T-cell function [39]. In the studies presented, expansion of CD8 T cells with cytolytic phenotype or function was observed with the combination of ixabepilone and anti-mCTLA-4 mAb in the EMT-6 mammary tumor model as well as with the pairing of etoposide and CTLA-4 blockade in the CT-26 colon carcinoma model. However, the precise mechanisms associated with the synergistic effects observed in the present studies were not fully characterized, and mechanistic studies will be required to identify the role of different cellular subsets in the generation of effective antitumor immunity.

In the clinic, ipilimumab has been evaluated in combination with dacarbazine in melanoma [27] or with paclitaxel/carboplatin in melanoma and lung cancer [40–42]. These clinical studies were designed to compare the activity of an ipilimumab-containing regimen to that of a chemotherapy control [27, 40–42]. Data from these clinical studies [27, 40–42] demonstrated improvement in overall survival with the combination regimen versus dacarbazine alone or improvement in immune-related progression-free survival in combination with paclitaxel/carboplatin. However, it is difficult to compare the results of the experiments presented in this article to these clinical studies since different chemotherapies and schedules were applied. Since the effect of distinct chemotherapeutic agents on tumor cell killing and/or immune function varies among agents, there are a myriad of possibilities in terms of schedules, doses, partnering agents, and tumor models that may be examined in future studies.

Overall, the data presented illustrate how a dual approach utilizing chemotherapy and anti-mCTLA-4 mAb can enhance antitumor effects over either agent alone in certain murine cancer models. It is important to note that the optimal schedule and dose to produce such antitumor effects in these murine models are not directly translatable to studies in humans. Furthermore, the impact of individual chemotherapies on the immune system and on their ability to kill tumor cells vary from each other, and these differences may be important in guiding choice of agents when combining chemotherapy with immunotherapy to optimize clinical outcomes. The data herein support the validity of combining chemotherapy with CTLA-4 blockade to mediate tumor regression and suggest further clinical study of these regimens for the treatment of cancer is warranted.
